# Transfer of Downy Mildew Resistance Genes from Wild Cucumbers to Beit Alpha Types

**DOI:** 10.3390/jof11080597

**Published:** 2025-08-16

**Authors:** Rivka S. Hammer, Yariv Ben Naim, Arnon Brand, Yigal Cohen

**Affiliations:** 1 Faculty of Life Sciences, Bar-Ilan University, Ramat Gan 5290000, Israel; rivka.sh.h@gmail.com (R.S.H.); yar2710@gmail.com (Y.B.N.); 2 Genesis Seeds, Ashalim 8500000, Israel; arnonbrand@gmail.com

**Keywords:** Cucurbitaceae, *Cucumis sativus*, cucurbit downy mildew, *Pseudoperonospora cubensis*, vegetable breeding, disease resistance

## Abstract

Downy mildew, caused by the oomycete *Pseudoperonospora cubensis*, is the most destructive foliar disease of cucumbers. While partially resistant slicer cultivars (with spined fruits) are commercially available, no resistant Beit Alpha cultivars (characterized by smooth, dark green fruit) have been developed to date. Here, we report the successful breeding of downy mildew-resistant Beit Alpha cucumber lines. Resistance was transferred from the wild Sikkim cucumber accessions PI 197088 and PI 330628 (characterized by round fruit, with heavily netted brown rind). The resistance and fruit phenotype were restored through backcrosses to elite commercial susceptible cultivars. Due to the recessive nature of the resistance genes and their distribution across multiple chromosomes, the breeding program required multiple backcrosses and stringent selections for both resistance and fruit type.

## 1. Introduction

Cucurbit downy mildew, caused by *Pseudoperonospora cubensis* (Berk. & Curt.) Rostov., is the most devastating foliar disease of cucumber, infecting a wide range of cucurbit species worldwide. Under natural conditions, cucurbit downy mildew affects most of the economically important species of the Cucurbitaceae family including cucumber (*Cucumis sativus* L.), cantaloupe (*Cucumis melo* L.), squashes and pumpkins (*Cucurbita* spp.), and watermelon (*Citrullus lanatus*) [1].

The pathogen produces angular chlorotic lesions, with purplish-black sporangia on the lower leaf surface. The sporangia disseminate via wind or water, initiating secondary infections. Infected foliage rapidly becomes necrotic, leading to premature plant death [2,3,4].

In the United States, *P. cubensis* sporangia are carried northward from overwintering sites in southern Florida by prevailing wind currents [5,6]. More recently, greenhouses in colder regions and oospore formation have been suggested as new inoculum sources [7]. Additionally, seed transmission has been proposed [8].

The pathogen is heterothallic, with A1 and A2 mating types. Isolates of pathotype 3 (P3, affecting cucumber and melon) are A1, while pathotype 6 (P6, affecting squash and pumpkin) includes both mating types [9]. Laboratory studies have shown profuse sporulation of A1P3 isolates on cucumber, moderate on melon, and slight sporulation on bottle gourd. They produced HR-like symptoms with no sporulation in the detached leaves of butternut gourd, squash, or pumpkin. In contrast, A2P6 isolates were compatible with all six plant species. A2P6 isolates have a broader host range but a lower rate of multiplication as compared with A1P3 isolates. Infection capacity is an important fitness parameter: one sporangium of A1P3 isolates is sufficient to produce a lesion in cucumber, while ten sporangia are required for A2P6 isolates [10].

Co-inoculation with A1 and A2 sporangia on detached leaves resulted in oospore formation in the mesophyll. Cucumber and melon enabled oospore production because they are compatible with both A1P3 and A2P6 isolates. However, squash, pumpkin, and butternut gourd are incompatible with A1P3 isolates, and therefore produce no oospores upon co-inoculation with A2P6 isolates [9]. These oospores produced F1 lesions when re-inoculated, with variable infectivity across cucurbit species [11]. Field reports of oospores have come from India [12] and the USA [13].

Globally, *P. cubensis* populations comprise two genetic clades. Clade 1, native to North America, infects most cucurbits except cucumber cultivars carrying the *dm1* gene. Clade 2, introduced to North America in 2004, preferentially infects cucumber and melon [14,15]. While Clade 1 isolates are native to North America, Clade 2 isolates (non-infective to *Cucurbita*) are native to Israel [10].

Major shifts in the global *P. cubensis* population structure were marked by two events: the emergence of Clade 1 (A2 mating type) in Israel in 2002 [16], and Clade 2 in the USA in 2004 [7]. The latter overcame the *dm1* resistance gene that had provided durable protection for decades. Clade 2 likely originated in East Asia [14], and its dissemination may have been facilitated by seed transmission [8]. Notably, current *P. cubensis* isolates in Israel show increased heat tolerance [17].

Host specificity plays a significant role in shaping pathogen populations. Clade 2 isolates primarily infect *Cucumis sativus*, *Cucumis melo*, and *Lagenaria siceraria*, while Clade 1 dominates on *Cucurbita* species, *Citrullus lanatus*, and *Momordica* spp. [15]. Similar host–pathotype relationships were reported in Israel [11]. Interestingly, watermelon in Israel remains unaffected, though it may develop necrotic lesions when near infected cucurbits. Clade-specific differences in fungicide response have also been observed [18].

The classical sources of downy mildew resistance in melon (*Cucumis melo*) are PI 124112 and PI 124111F. The latter carries two semi-dominant genes *Pc1* and *Pc2* [19] that encode for At1 and At2, two enzymes that catalyze the synthesis of aminotransferases via the formation of reactive oxygen species [20].

The classic source of downy mildew resistance in cucumber PI 197087 carries the recessive gene *dm1*, which triggers a hypersensitive response (HR) upon infection [21,22,23]. Though once highly effective, this gene’s resistance was compromised after 2004 by new *P. cubensis* strains [7,24]. Nonetheless, *dm1* still confers moderate resistance in the USA and retains high efficacy elsewhere [25].

The need for novel sources of resistance became urgent after 2004. Sikkim-type cucumber accessions PI 197088 and PI 330628 were identified as promising new sources, each harboring multiple QTLs for resistance to both downy and powdery mildew [26,27,28,29]. In these lines, inoculated leaves develop small, HR-like lesions with limited sporulation, and are strongly associated with callose and lignin deposition [10,30].

However, the fruit of Sikkim cucumbers—round, 15–20 cm in diameter, with a rough brown rind—are unsuitable for commercial markets. Crosses between the Sikkim PIs and elite susceptible lines yielded F1 plants with elongated brown fruits and susceptibility [27]. In the F2 generation, resistance and fruit traits segregated, and only ~1 in 164 plants exhibited a resistance level matching the Sikkim parents, indicating a polygenic, recessive inheritance [30]. QTL mapping identified multiple resistance loci on various chromosomes of these PIs [31,32,33].

In response to the breakdown of *dm1*-mediated resistance, breeding programs introduced new resistant cultivars. Cornell University developed ‘DMR-NY264’, derived from the ‘Marketmore’ and ‘Poinsett’ series [34]. While its precise genetic basis remains unclear, it likely benefits from additive resistance traits. However, its delayed fruiting made it less ideal for shorter growing seasons. An improved derivative, ‘DMR-NY401’, was developed by crossing ‘DMR-NY264’ with ‘Dasher II’, producing earlier, more prolific plants while retaining resistance [35]. In field trials conducted in LIHREC, ‘TSX-CU231AS’ and ‘DMR-NY264’ showed the highest resistance to downy mildew and produced the highest yields among tested varieties. ‘Brickyard’ and ‘Bristol’ exhibited moderate to high resistance, and a higher yield than the susceptible variety Speedway [36].

In China, the cucumber inbred line K8 has been used to study the genetic control of DM resistance in order to provide a theoretical basis for the resistance mechanisms and for marker-assisted selection (MAS) [37]. The old Japanese cultivar Santou exhibits moderate resistance in different plant organs (cotyledons, true leaves) and stages (seedlings and adult plants) [33]. In South Korea, TH118FLM, an advanced self-pollinating inbred line derived from the commercial hybrid variety ‘Malini’ (Monsanto), has been used to detect QTLs for DM resistance [38].

Recently, new sources of partial resistance to both downy and powdery mildew were identified in non-Sikkim cucumber accessions from East Asia [39].

The objective of this study was to transfer the genes for resistance against downy mildew from the wild inedible Sikkim cucumbers PI 197088 and PI 330628 to modern, otherwise susceptible, cultivars to produce dark green, smooth-skinned, medium-length fruits of the Beit Alpha type.

## 2. Materials and Methods

### 2.1. Plant Sources

Seeds of PI 197088, PI 330628, and SMR-18 were obtained from the USDA. Seeds of Dlila F1, Romi F1, and Nena F1 were provided by Hazera Seeds, Israel. Seeds of the commercial cultivars Katrina F1 and Deluxs F1 were provided by Genesis Seeds, Ashalim, Israel.

### 2.2. Agronomy

Breeding, inoculation, and evaluation activities were conducted between 2016 and 2025 at Bar-Ilan University, Ramat Gan, Israel (32.1002° N, 34.8502° E) and at the Experimental Farm of Genesis Seeds, Ashalim, Israel (31.4276° N, 34.5882° E).

Plants were grown in 120 L containers, 8 plants per container, arranged in 4 rows of 30 containers (total 960 plants per net house) in a high net house (50 × 9 × 4.5 m) covered with 50-mesh anti-insect white plastic sheets. Two houses were cultivated per growing season, with two growing seasons per year (spring and autumn), totaling approximately 2000 plants per season. Containers were filled with a peat/perlite mixture (10:1, *v*/*v*), and plants were irrigated via drip systems with 0.5% N:P:K fertilizer solution.

Cucumber seeds were sown in multicell trays in a commercial nursery and transplanted into the net houses at the 2-leaf stage.

Breeding and selection were conducted twice annually: spring season: planting in late March, seed harvest in late June, and autumn season: planting in early September, seed harvest in mid-December.

At the 7–10-leaf stage, after being inoculated with downy mildew, plants were supported vertically using trellising. Crosses were performed manually. Femaleness was induced when needed using a single foliar spray of 0.05% AgNO_3_.

### 2.3. Breeding

**Figure 1:**
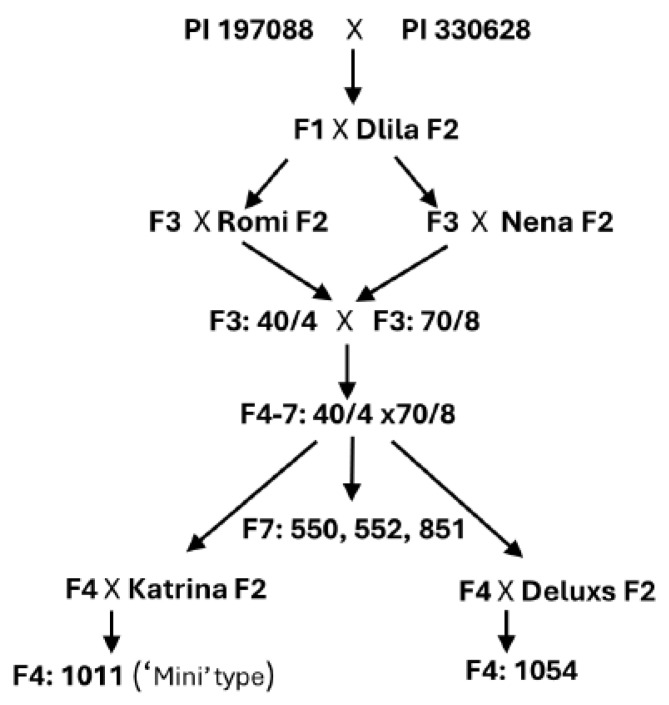
Pedigree scheme of five downy mildew-resistant Beit Alpha cucumber lines. *Cucumis sativus* PI 197088 and PI 330628 are Sikkim types, highly resistant to downy mildew. They were crossed with the commercial Beit Alpha cultivars Dlila, Romi, Nena, Katrina, and Deluxs to produce resistant Beit Alpha types.

A flow chart showing our breeding procedures is shown in Figure 1. Based on our previous study [27], which indicated that some downy mildew (DM) resistance genes in PI 197088 and PI 628330 are likely non-allelic, a cross between these two lines was initiated after three generations of self-pollination to ensure homozygosity. The resulting F1 plants of (197088 × PI 330628) were crossed with the commercial, DM-susceptible Beit Alpha Dlila F2 plants and their descendant F3 plants were crossed with the commercial, DM-susceptible Beit Alpha type Romi F2 or DM-susceptible Beit Alpha type Nena F2. The F3 descendants of Romi (40/4) were crossed with the F3 descendants of Nena (70/8). Seven cycles of self-pollination of (40/4 × 70/8) resulted in the creation of three Beit Alpha-resistant lines, 550, 552, and 851. The F4 descendants of (40/4 × 70/8) were crossed with the DM-susceptible elite Beit Alpha type Katrina F2 to produce the Mini type of Beit Alpha 1011. A similar cross was performed with the elite DM-susceptible Beit Alpha Deluxs F2 to produce Beit Alpha 1054.

### 2.4. Inoculation

About three weeks after transplanting, when plants reached the 6–7-leaf stage, they were inoculated with *Pseudoperonospora cubensis*, the causal agent of downy mildew. Inoculation was performed at sunset using a hand sprayer to apply ~5 mL per plant of a sporangial suspension (2000 sporangia/mL) to the upper leaf surfaces. Plants were then covered overnight with plastic sheets to ensure high humidity and promote infection.

The inoculum consisted of a mixture of *P. cubensis* isolates: pathotype 3 (A1 mating type, Clade 2) and pathotype 6 (A2 mating type, Clade 1), as previously described [10,30,39]. The pathogen was maintained on infected cucumber leaves and stored at −80 °C.

### 2.5. Resistance Ratings

Disease assessments began one week after inoculation and were conducted every 7–10 days. For each plant, the percentage of leaf area affected by downy mildew was visually estimated. Lesion types were recorded, and sporulation intensity on the abaxial leaf surface was visually evaluated. In this breeding program, a plant was considered highly resistant if lesions covered ≤5% of the leaf area, lesion size was <2 mm, and no visible sporulation of *P. cubensis* was observed. The area under disease progress curve (AUDPC) was calculated based on the disease records gathered during the season. A cucumber line displaying a mean AUDPC lower by 90% compared to the susceptible control was considered highly resistant.

## 3. Data Analysis

A total of 1920 plants were grown per season, 8 plants per entry, two growing seasons per year (spring and autumn). Disease records, which were taken 5–6 times during a season at intervals of 7–10 days, were used to calculate the AUDPC (area under disease progress curve) values. The mean AUDPC values were tested for normality and variance homogeneity using the Shapiro test and Levene test, which showed no normal distribution and no variance homogeneity. The Kruskal test was performed following Dunn’s test using R 4.5.0 software.

## 4. Results

### 4.1. Disease Resistance

**Figure 2:**
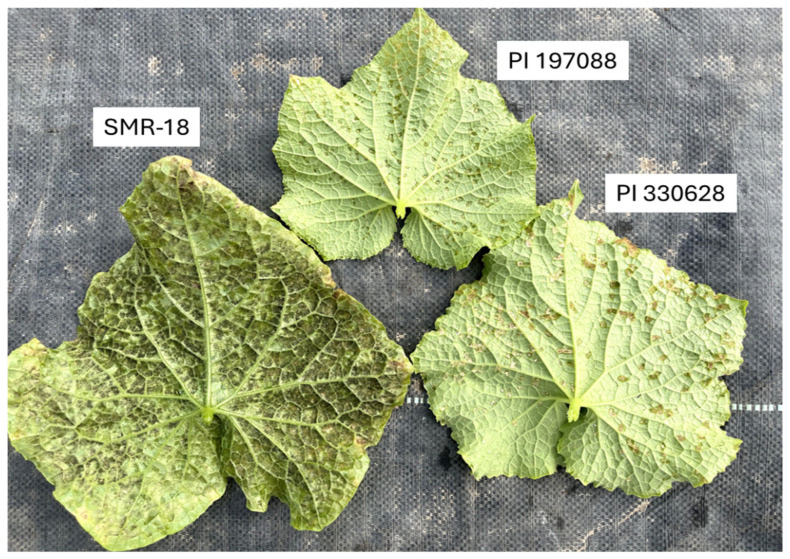
Lesion type and sporulation of downy mildew caused by *Pseudoperonospora cubensis* on the adaxial (lower) surface of the susceptible SMR-18 and the resistant PI 197088 and PI 330628. Note heavy sporulation on SMR-18 (105,000 ± 3500 sporangia/cm^2^), pinpoint water-soaked lesions in PI 197088 (10 ± 10 sporangia/cm^2^), and small necrotic lesions in PI 330628 (120 ± 30 sporangia/cm^2^). The photo was taken 10 days after artificial inoculation in the net house. Scale bar = 10 cm.

Ten days after inoculation, the susceptible control (SMR-18) developed large (7–12 mm) chlorotic lesions with abundant sporulation on the lower leaf surface, whereas PI 197088 exhibited pinpoint (~0.5 mm) water-soaked lesions, and PI 330628 developed small (~1 mm), water-soaked necrotic lesions. Both accessions showed no visible sporulation (Figure 2).

**Figure 3:**
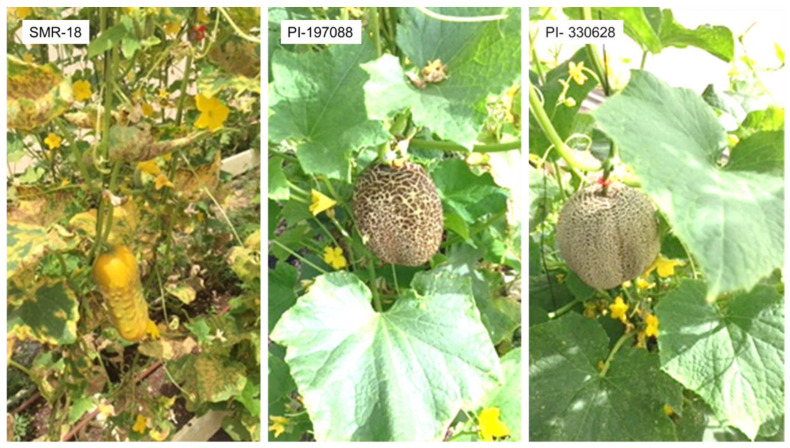
The appearance of downy mildew caused by *Pseudoperonospora cubensis* in mature plants of the susceptible SMR-18 and the resistant PI 197088 and PI 330628. Note the Sikkim-type fruit and high resistance to downy mildew in PI 197088 and PI 330628. The photo was taken 60 days after artificial inoculation in a net house. Scale bar = 15 cm.

At fruit maturity, SMR-18 plants were heavily affected, with widespread leaf desiccation. PI 197088 remained mostly lesion-free, while PI 330628 showed a few lesions on the middle leaves and none on the upper leaves (Figure 3). The results support our previous findings [30,39] indicating high resistance of both PIs to multiple *P. cubensis* isolates.

The F1 plants of the cross (PI 197088 × PI 330628) were highly resistant and bore round Sikkim-type fruit [30,39]. These F1 plants were crossed with the susceptible Beit Alpha commercial Dlila F1 after self-pollination. The F1 progeny plants of [PI 197088 × PI 330628) × Dlila F2] displayed partial resistance and oblong Sikkim-type fruits.

**Figure 4:**
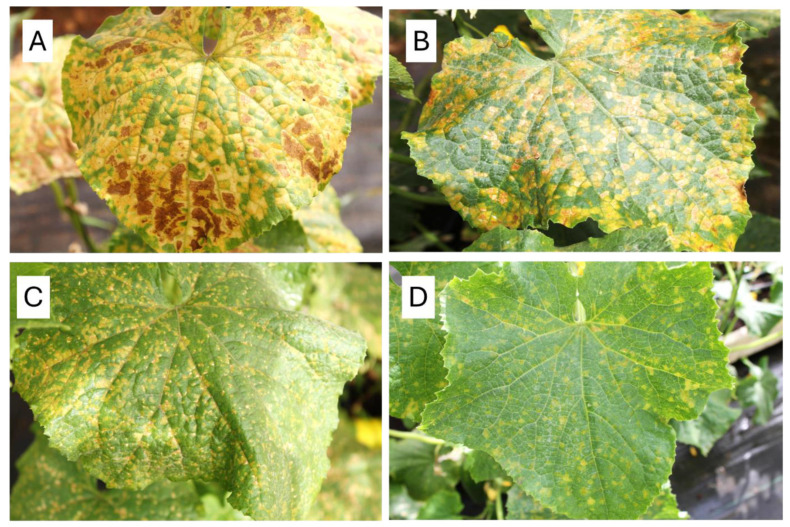
Symptoms of downy mildew caused by *Pseudoperonospora cubensis* on F2 cucumber plants segregating for resistance of the cross [PI197088 × PI 330628) × Dlila F2]) (and their associated AUDPC values at 59 dpi). (**A**) Highly susceptible (AUDPC = 3300 ± 820). (**B**) Susceptible (AUDPC = 2200 ± 650). (**C**) Moderately resistant (AUDPC = 850 ± 240). (**D**) Resistant (AUDPC = 300 ± 45).

Self-pollination of these F1 plants produced a few highly resistant F2 individuals with non-Sikkim fruit types. Lesion types observed in the F2 population of the cross [PI 197088 × PI 330628) × Dlila F2] are illustrated in Figure 4, ranging from angular, large chlorotic lesions with sporulation to tiny necrotic spots with no sporulation.

**Figure 5:**
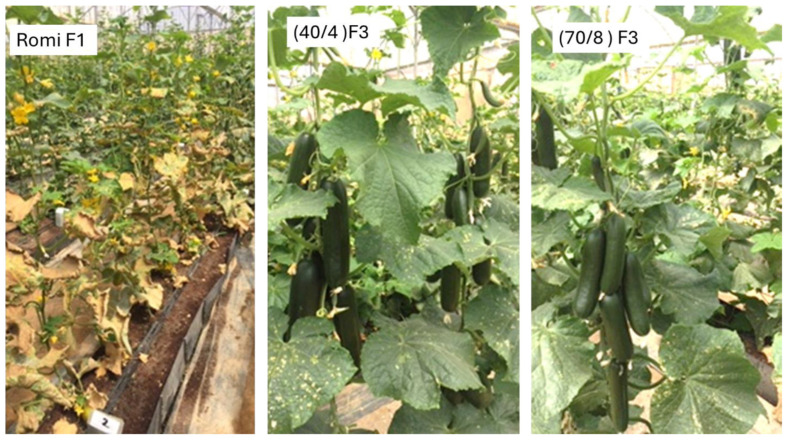
The appearance of Romi F1, a commercial cucumber cultivar susceptible to downy mildew (**left**), and the resistant lines 40/4 F3 (**middle**) derived from a backcross to Romi F2, and 70/8 F3 derived from a backcross to Nena F2 (**right**). Note: The heavy infection in Romi F1 as against almost no infection in 40/4 and 70/8. Also, note the Beit Alpha fruit type and good yield. The photo was taken at Ashalim, 50 days after inoculation.

The few highly resistant F2 plants were self-pollinated, and their F3 progeny plants were selected for high resistance and Beit Alpha fruit type and backcrossed to the elite but susceptible Beit Alpha cultivars Romi F2, producing line 40/4, or to Nena F2, producing line 70/8 (see Figure 1). The appearance of line 40/4 = {[PI 197088 × PI 330628) × Dlila F2] × Romi F2} and line 70/8 = {[PI 197088 × PI 330628) × Dlila F2] × Nena F2} is shown in (Figure 5). Both exhibited high resistance and good fruit quality.

**Figure 6:**
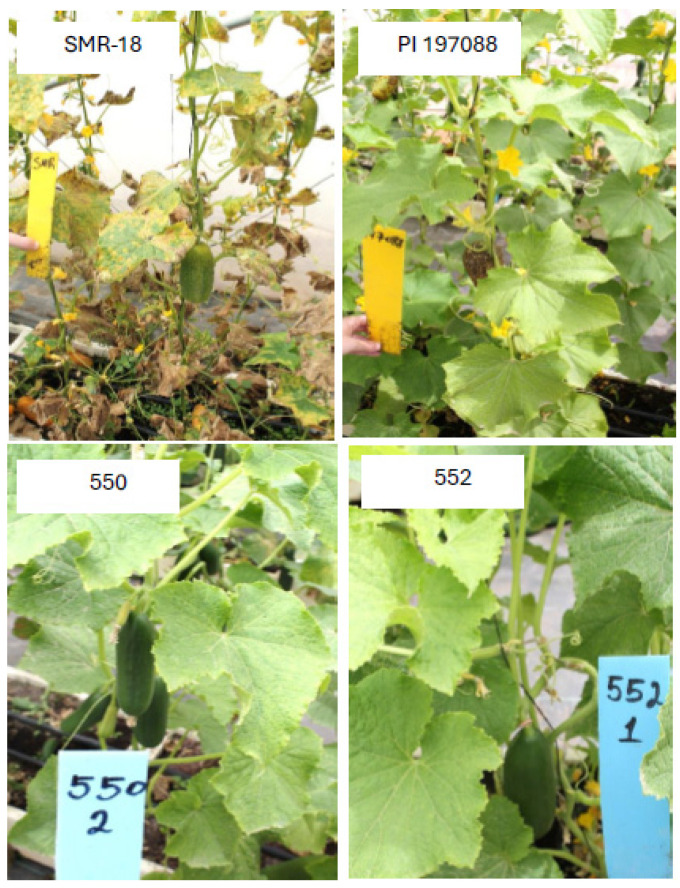
Appearance of downy mildew on the susceptible SMR-18 and the resistant PI 197088. Lines 550 and 552 are highly resistant F7 descendants of the cross 40/4 × 70/8.

**Figure 7:**
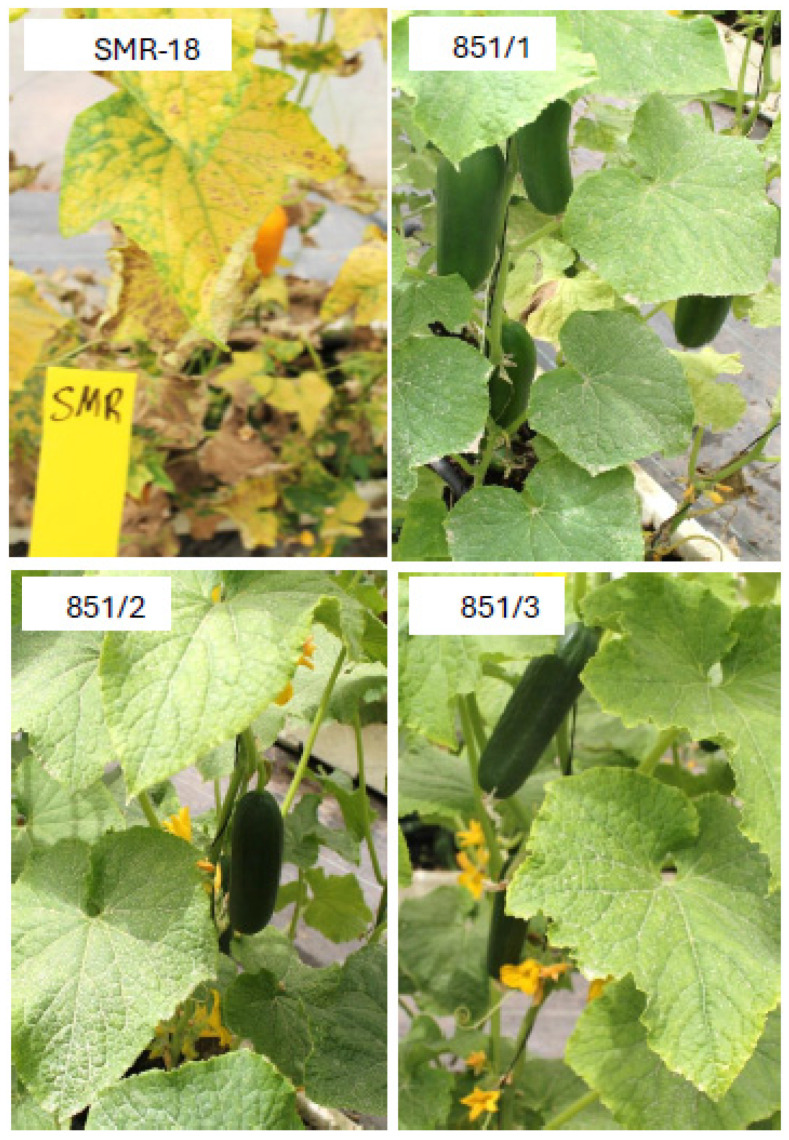
Three Beit Alpha cucumber lines are highly resistant to downy mildew. Lines 851/1, 851/2, and 851/3 are F7 descendants of the cross (40/4 × 70/8) F2.

Lines 40/4 and 70/8 were crossed, and the F1 progeny plants of (40/4 × 70/8) were self-pollinated for six generations (Figure 1) to achieve homozygosity in resistance, femaleness, earliness, parthenocarpy, and fruit quality, culminating in the production of lines 550 and 552 (Figure 6), and line 851 (Figure 7), all with a Beit Alpha fruit type and displaying high resistance to downy mildew.

F1 plants from the backcross [40/4 × 70/8) × PI 330628] exhibited high resistance, confirming the homozygosity of resistance in both parents.

**Figure 8:**
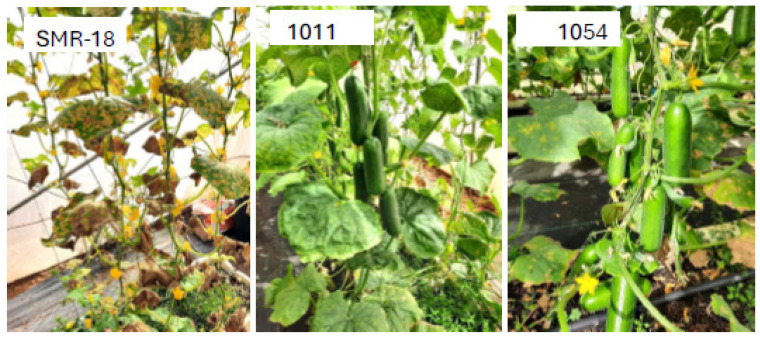
Downy mildew-resistant lines 1011 and 1054. See Figure 1 for their pedigree. Note the severe disease in the susceptible control SMR-18. The photo was taken at Bar Ilan University 59 days after inoculation.

F4 descendants of (40/4 × 70/8) were crossed with Katrina F2 and with Deluxs F2 (see Figure 1), resulting in highly resistant high-quality Beit Alpha lines 1011 (Mini type) and 1054 (Figure 8).

Table 1 summarizes the mean response to downy mildew of the commercial, bred, and PI cucumber entries during the spring season of 2025. Ten commercial cultivars were highly susceptible with a mean AUDPC value of 3269 ± 776. All the newly bred lines, as well as the DMR 401 of Cornell, were significantly more resistant to the disease, displaying much smaller mean AUDPC values of 236–875 (Table 1). The resistant wild parents PI 197088 and PI 330628 exhibited the lowest mean AUDPC values of 92 and 272, respectively. Among the five resistant pedigrees that were developed in this study, lines (40/4 × 70/8) #1 and (40/4 × 70/8) #5 were the most resistant, displaying the smallest AUDPC values of 249 and 236, respectively. Interestingly, the F1 hybrid of [(40/4 × 70/8 #3) × DMR 401 F2] was more resistant than its parents, suggesting complementation of some non-allelic resistance genes. The mean AUDPC values presented by our top resistant lines 552 and 851 were 92.4% and 92.8% lower, respectively, compared to the commercial cultivars, whereas those of their parents PI 330628 and PI 197088 were 91.7% and 97.2% lower, respectively, than the commercial cultivars, indicating efficient introgression of the resistance genes from the wild PIs into the cultivated cucumbers.

### 4.2. Performance of the Resistant Lines

Lines 550, 552, and 851 are Beit Alpha-type cucumbers, with medium fruit length (12–14 cm), smooth, shiny dark green skin. These lines are gynoecious and parthenocarpic, typically producing 2–3 female flowers per node. The first female flowers appear around the fourth–fifth internode, approximately 25 days after planting. The first harvest occurs about 45 days after planting. They maintained high resistance to downy mildew throughout the growing season until seed maturity (95 days after planting, 76 days after disease onset).

Line 1011 is a descendant of the Mini Beit Alpha type Katrina F1 with 10–12 cm long dark green fruits, whereas line 1054 is a descendant of the Beit Alpha type Deluxs F1 with 14–16 cm long dark green fruits. They are high yielders, gynoecious, and parthenocarpic, exhibiting good resistance to downy mildew.

## 5. Discussion

Downy mildew is a major limiting factor in cucumber production worldwide. The recently evolved new global populations of the causal agent *Pseudoperonospora cubensis* have become more aggressive, fungicide-resistant, and heat-tolerant [7,16,17,18].

In the United States, the emergence (or possible migration) of this new pathogen population has compromised the effectiveness of the widely used resistance gene *dm1* [7,24]. Similarly, in Israel, novel populations have begun infecting *Cucurbita* species that were previously unaffected [16].

Global efforts to breed cucumbers for resistance against downy mildew have so far yielded only a limited number of resistant slicer-type cultivars [33,34,35,37,38]. In a recent review article, Mirzwa-Mroz et al. described the genetic basis of downy mildew resistance in cucumbers, emphasizing key resistance (R) genes and quantitative trait loci (QTLs) that have been mapped, as well as recent advances in molecular breeding tools, including marker-assisted selection (MAS), genomic selection (GS), and CRISPR/Cas9 genome editing that have accelerated the development of resistant cultivars of the slicer type [40]. However, there is strong market demand for dark green, smooth-skinned, medium-length cucumbers of the Beit Alpha type.

The original Beit Alpha cucumber was bred in Israel in 1931 by the horticulture expert Hanna Lazarson at Kibbutz Beit Alpha from the landrace variety Damascus that was grown in the region. High-yield Beit Alpha types, with enhanced shelf life, were bred later by Hazera Seeds, Vilmorin, and BASF, but no downy mildew resistance was incorporated. Most Beit Alpha-type varieties develop multiple fruits (2–4) at every node, throughout the season. Fruits are selectively picked when reaching 15–20 cm long (70–100 g per fruit). A plant may produce 50–70 fruits in a greenhouse (14–21 kg/m^2^). Harvested fruits can be stored at 10–14 °C for up to 14 days [41,42,43].

The breeding program described in this study was specifically aimed at developing downy mildew-resistant Beit Alpha cucumbers.

The most well-known sources of resistance, PI 197088 and PI 330628, possess two significant drawbacks: (a) resistance is conferred by multiple recessive alleles; and (b) these accessions also carry semi-dominant genes responsible for undesirable Sikkim-type fruits, characterized by a round shape and heavily netted brown rind [10,26,27,28,29,30,39].

As demonstrated in this study, the introgression of resistance genes from such wild sources into cultivated lines required multiple cycles of backcrossing to the elite, susceptible cultivars, along with careful selection for both disease resistance and desirable fruit type.

These intensive efforts, which spanned a decade, succeeded in producing cucumber lines that combine the resistance traits of the PI sources with the fruit quality characteristics of elite Beit Alpha cultivars. Before these lines are released to market, future field trials or natural infection testing across multiple environments or years are planned. This would help confirm the durability of resistance under diverse real-world conditions and the possibility of negative trade-offs associated with resistance introgression—such as reduced plant vigor or increased susceptibility to other pathogens—which are commonly encountered in breeding programs.

## 6. Conclusions

This long-term breeding study has culminated with the development of cucumber lines resistant to the newly emerging populations of the downy pathogen *P. cubensis*. Unlike slicers and pickling cucumbers, the new lines belong to the Beit Alpha type that are characterized by dark green, smooth, medium-length fruits. The new lines fulfil the strong expectations of the market.

## Figures and Tables

**Table 1:** Downy mildew development on commercial cucumber cultivars as compared to resistant lines produced in this study. AUDPC values (area under disease progress curve) were collected during spring 2025, 59 days after artificial inoculation with *Pseudoperonospora cubensis*. Different letters under the Dunn’s test column indicate significant difference between means at α = 0.05.

			AUDPC			
Pedigree (Code)	Fruit Type	Replicates	Mean	Std Dev	Dunn’s Test	% Reduction
Ten commercial cultivars	Various	104	3269	776	a	0
[(40/4 × 70/8 #5) × Katrina F2] F4 (1054)	Beit Alpha	32	875	261	b	73.2
[(40/4 × 70/8 #5) × Deluxs F2] F4 (1011)	Beit Alpha	32	855	255	b	73.8
40/4 × 70/8 #3 (550)	Beit Alpha	32	830	244	b	74.6
DMR 401 F2	Slicer	16	536	145	bc	83.6
[(40/4 × 70/8 #3) × DMR 401 F2] F1	Slicer	16	279	306	c	91.5
PI 330628	Sikkim	32	272	46	c	91.7
[(40/4 × 70/8 #3) × PI 330628] F1	Sikkim	8	256	274	bc	92.2
40/4 × 70/8 #1 (851)	Beit Alpha	24	249	199	c	92.4
40/4 × 70/8 #5 (552)	Beit Alpha	24	236	190	c	92.8
PI 197088	Sikkim	16	92	96	c	97.2

## Data Availability

The original contributions presented in this study are included in the article. Further inquiries can be directed to the corresponding author.
